# The regulatory and predictive functions of miR-17 and miR-92 families on cisplatin resistance of non-small cell lung cancer

**DOI:** 10.1186/s12885-015-1713-z

**Published:** 2015-10-19

**Authors:** Jian Zhao, Wenfan Fu, Hongying Liao, Lu Dai, Zeyong Jiang, Youguang Pan, Haoda Huang, Yijun Mo, Siwen Li, Guangping Yang, Jun Yin

**Affiliations:** Department of Chest Surgery, Cancer Center of Guangzhou Medical University, Guangzhou, Guangdong China

**Keywords:** Non-small cell lung cancer, miR-17 and miR-92 families, Cisplatin resistance, G1 phase arrest, DNA repair

## Abstract

**Background:**

Chemotherapy is an important therapeutic approach for non-small cell lung cancer (NSCLC). However, a successful long-term treatment can be prevented by the occurring of chemotherapy resistance frequently, and the molecular mechanisms of chemotherapy resistance in NSCLC remain unclear. In this study, abnormal expressions of miR-17 and miR-92 families are observed in cisplatin-resistant cells, suggesting that miR-17 and miR-92 families are involved in the regulation of cisplatin resistance in NSCLC.

**Methods:**

miRNA microarray shows that miR-17 and miR-92 families are all down-regulated in cisplatin-resistant A549/DDP cells compared with cisplatin-sensitive A549 cells. The aim of this study is to investigate the regulatory functions of miR-17 and miR-92 families on the formation of cisplatin resistance and the predictive functions of them as biomarkers of platinum-based chemotherapy resistance in NSCLC.

**Results:**

The low expressions of miR-17 and miR-92 families can maintain cisplatin resistance through the regulation of *CDKN1A* and *RAD21*. As a result of high expressions of *CDKN1A* and *RAD21*, the inhibition of DNA synthesis and the repair of DNA damage are achieved and these may be two major contributing factors to cisplatin resistance. Moreover, we demonstrate that the expressions of miR-17 and miR-92 families in NSCLC tissues are significantly associated with platinum-based chemotherapy response.

**Conclusion:**

Our study indicates that miR-17 and miR-92 families play important roles in cisplatin resistance and can be used as potential biomarkers for better predicting the clinical response to platinum-based chemotherapy in NSCLC.

**Electronic supplementary material:**

The online version of this article (doi:10.1186/s12885-015-1713-z) contains supplementary material, which is available to authorized users.

## Background

Lung cancer is the highest morbidity and mortality cancer in the world, and about 80 % cases are non-small cell lung cancer (NSCLC). In NSCLC, about 70–80 % cases are diagnosed at advanced stage and lost the opportunity of surgery, and chemotherapy is the main treatment for these cases. However, by using chemotherapy, the long term survive rate of NSCLC is only 10–15 %, and chemotherapy resistance is one of the main reasons leading to the failure of chemotherapy [1, 2]. Although numerous mechanisms of chemotherapy resistance have been described, it is still not fully elucidated at present [3–5]. Therefore, the research of chemotherapy resistance is important for the successful chemotherapy of NSCLC.

MicroRNA (miRNA) is a kind of endogenous small non-coding RNA, and it has been found that miRNAs play important roles in the regulation of gene expression at the post-transcriptional level through base pairing to the 3’ untranslated region (3’-UTR) of target messenger RNA (mRNA) [6]. Currently, more than 1000 miRNAs among human genome have been discovered. miRNAs are involved in a variety of basic biological processes, and a lot of miRNAs have been found can affect the development of cancer as oncogenes or tumor suppressor genes depending on their targets [7–9]. The miR-17 ~ 92 cluster and paralogous, which are recognized as typical oncogene-type miRNAs, have been shown to promote tumorigenesis of many cancer types, such as lung cancer, medulloblastoma, leukaemia, hepatocellular carcinoma and so on [10–12].

Cisplatin is a very common chemotherapeutic drug for anticancer chemotherapy. In general, cisplatin-induced cytotoxicity can trigger the cellular apoptosis and produce therapeutic effects [13]. However, during chemotherapy with cisplatin, it tends to develop cisplatin resistance frequently as a result of the defects in cellular functions, such as drug absorption, cell proliferation, cell apoptosis, DNA repair, and other biological process still unknown [14]. Recently, miRNAs also have been found play important roles in chemotherapy resistance [15–19]. Here, we find that both miR-17 and miR-92 families, two families of miR-17 ~ 92 cluster and paralogous, are down-regulated expressions in cisplatin-resistant A549/DDP cells compared with cisplatin-sensitive A549 cells. The low expressions of miR-17 and miR-92 families can result in the high expressions of target genes *CDKN1A* (cyclin-dependent kinase inhibitor 1A) and *RAD21* (Rad21 homolog (S. pombe)). CDKN1A can arrest cells at G1 phase and RAD21 can enhance the DNA repair, so that A549/DDP cells can avoid cisplatin-induced cytotoxicity by reducing DNA synthesis and DNA damage. Moreover, the low expressions of miR-17 and miR-92 families in NSCLC tissues are associated with platinum-based chemotherapy resistance. In a word, our results demonstrate that both miR-17 and miR-92 families are involved in the regulation of cisplatin resistance and may have potential as new biomarkers for predicting platinum-based chemotherapy resistance in NSCLC.

## Methods

### Cell lines and cell culture

Human non-small-cell lung cancer cells A549 (parental) and A549/DDP (cisplatin-resistant) were cultured in RPMI 1640 medium supplemented with 10 % fetal bovine serum at 37 °C in a humidified atmosphere with 5 % CO_2_. A549/DDP cells were induced by using progressive concentration of cisplatin as described previously [20]. Cisplatin (1 μg/ml) was also added for the culture of A549/DDP cells.

### Tissue sample collection

NSCLC tissues were collected from the Cancer Center of Guangzhou Medical University (Guangzhou, China) with informed consent and Institutional Review Board (IRB) permission. Thirty-nine NSCLC patients were recruited for this study. All of the following criteria were met: patients who suffered from primary NSCLC; a histological diagnosis of NSCLC with at least one measurable lesion; a TNM clinical stage of IIIB to IV; first-line chemotherapy with platinum-based chemotherapy every 3 weeks for a maximum of 4 cycles. Fresh NSCLC tissues were obtained by aspiration biopsy before chemotherapy and immediately snap-frozen in liquid nitrogen. NSCLC tissues were stored in liquid nitrogen until use. All clinical and biological data were available for the samples.

According to the RECIST (Response Evaluation Criteria in Solid Tumors), tissue samples were divided into two groups according to the patient’s responses assessed by medical image analysis and detection of serum tumor markers after 4 cycles of the platinum-based chemotherapy: response or partial response, at least 30 % decrease in the sum of diameters of target lesions from pre-chemotherapy levels, taking as reference the baseline sum diameters; stable or progressive disease, decrease of less than 30 % or increase from pre-chemotherapy levels, taking as reference the baseline sum diameters. Patients with chemotherapy response or partial response were considered as chemotherapy sensitivity (R, responder), whereas patients with stable or progressive disease were grouped together as chemotherapy resistance (NR, non-responder).

All patients provided written informed consent. This study was approved by the ethics committee of Cancer Center of Guangzhou Medical University (Approval no. (2014) 66).

### Microarray detection of miRNA expression

The microarray assay and result analysis were performed as described previously [20]. Briefly, the total RNA of A549 cells and A549/DDP cells were isolated by Total RNA Purification Kit. The microarray hybridization assays were carried out in two experimental repeats of the RNA samples obtained from A549/DDP cells (Cy5-labeled) and A549 cells (Cy3-labeled). Hybridization images were collected using a laser scanner and digitized using Array-Pro image analysis software. Data were analyzed by first subtracting the background and then normalizing the signals using an LOWESS filter.

### RNA extraction and RT-PCR

Total RNA of cells and tissue samples were extracted by Trizol (Invitrogen, USA) according to the manufacturer’s instruction. Quantitative real time polymerase chain reaction (RT-PCR) was performed by using ABI ViiATM7Dx Real-Time PCR System (Life Technologies, USA). For mRNA detection, 1 μg total RNA was used for cDNA synthesis using a Reverse Transcription Kit (Takara, Japan), then cDNA was used for RT-PCR using primers and SYBR Green Realtime PCR Master Mix (TOYOBO, Japan). For miRNA detection, 1 μg total RNA was used for cDNA synthesis using a miScript II RT Kit (Qiagen, Germany), then cDNA was used for RT-PCR using miScript Primer Assay (Qiagen, Germany) and miScript SYBR Green PCR Kit (Qiagen, Germany). Expression levels of mRNA and miRNA were normalized by β-Actin and U6 (Sigma, USA) separately.

The mRNA RT-PCR primers were designed as the following:

- *CDKN1A*: Forward 5’-TGCCGAAGTCAGTTCCTTGT-3’; Reverse 5’-CATTAGCGCATCACAGTCGC-3’.
- *RAD21*: Forward 5’-AGTGAATCGGAAAGGAGGCG-3’; Reverse 5’-ACTCTCCACGCTGCTCTCTA-3’.
- β-*Actin*: Forward 5’-GAGCACAGAGCCTCGCCTTT-3’; Reverse 5’-AGAGGCGTACAGGGATAGCA-3’.

### Dual luciferase report system detection of target genes

The wild type (WT) and mutant type (MUT) 3’-UTR of *CDKN1A* and *RAD21* were chemically synthesized and inserted into psiCHECK™ -2 vector (promega, USA) for getting psiCHECK™-2-*CDKN1A*-3’-UTR or psiCHECK™-2-*RAD21*-3’-UTR (WT or MUT). Dual luciferase analysis was performed as described previously [20]. Briefly, for miRNA mimics assay, 50 ng plasmids DNA were transfected in combination with 1.5 nmol of the miRNA mimics or notarget control (NC) using Lipofectamine Transfection Reagent (Invitrogen of life). For mRNA protector assay [21], the mRNA protectors for CDKN1A and RAD21 were designed as complementary sequences covering the miRNA binding sites in their 3’-UTRs, and they were inserted into the pCDNA3.1 vector for the luciferase assay in a 3:1 ratio (protector : miRNA mimic). In addition, the mRNA protectors for CDKN1A and RAD21 were inserted into the pCAGIG vector for the transfection assay in the subsequent. Luciferase activity was measured 48 h after transfection using the Dual Luciferase Reporter Assay System (Promega).

### The cytotoxicity influence of miRNAs

According to the manufacturer’s protocol, 100 nM of miRNA mimics, miRNA protectors, siRNAs or notarget control miRNAs (NC) were transfected into cells by Lipofectamine Transfection Reagent (Invitrogen of life).

Quantitative cytotoxicity analysis of cisplatin resistance level was determined by using CCK8 (CCK-8, Dojindo Laboratories, Japan) and represented in IC50 (μg/ml) as described previously [20]. Briefly, the suspensions of cells were incubated with different concentrations of cisplatin. After 48 h, after the medium with cisplatin were removed, RPMI1640 medium (Gibco, USA) of 90 μL and CCK-8 solution of 10 μL were added into each well of the plate. The cells on the plate were incubated for 3–6 h in the incubator. The absorbance at 450 nm wavelength was measured on an automated reader (TECAN, Switzerland).

### Western blotting

The cells were harvested at 72 h after transfection of 50 nM of miRNA mimics or miRNA protectors, and lysed by RIPA buffer for 30 min at 4 °C. 50 μg proteins were loaded into 15 % SDS–PAGE for analysis. The first antibody was rabbit polyclonal anti-CDKN1A or anti-RAD21 (purchased by cell signaling USA, 1:1000 dilutions). The secondary antibody was goat-anti-rabbit IgG conjugated with HRP (horseradish peroxidase) with a dilution of 1:1000. The bound antibodies were detected using ECL Plus Western Blotting Detection system (GE Healthcare). β-Actin was used as an internal control.

### Apoptosis analysis

A549/DDP cells were transfected with 50 nM of miRNA mimics, miRNA protectors or siRNAs in 60 mm petri dish. 24 h after transfection, cisplatin (2 μg/ml) was added to each petri dish and the cells were cultured for 24 h. According to the manufacturer’s protocol, cell apoptosis was detected using an Annexin V-FITC apoptosis detection kit (BD, USA) by flow cytometric analysis.

### Cell cycle assay

A549/DDP cells or A549 cells were transfected with 50 nM of miRNA mimics, miRNA protectors or siRNAs in 60 mm petri dish. 48 h after transfection, cells were stained with propidium iodide (PI) for cell cycle analysis by flow cytometry.

### EdU assay

A549/DDP cells or A549 cells were transfected with 50 nM of miRNA mimics, miRNA protectors or siRNAs in 96-well plates. 48 h after transfection, the cells were added by 100 mM EdU (5-ethynyl-2’-deoxyuridine) and cultured for an additional 2 h according to the protocol (Cell Light EdU DNA imaging Kit). Then, the cells were stained with Hoechst (5 mg/ml) for 30 min. Images were taken and analyzed by High Content Imaging Pathway 855 (BD, USA). EdU positive ratio was calculated by (EdU add-in cells/Hoechst stained cells) × 100 % [22].

### Immunofluorescent microscopy

A549/DDP cells or A549 cells were transfected with 50 nM of miRNA mimics, miRNA protectors or siRNAs in 6-well culture plate. 24 h after transfection, cells were treated with cisplatin (2 μg/ml) for 24 h, washed with PBS and fixed in 4 % paraformaldehyde for 10 min at room temperature. Fixed cells were washed with PBS, and permeabilized in 0.25 % Triton X-100 for 10 min. After treatment with 5 % BSA blocking agents for a minimum of 30 min at room temperature, samples were incubated with a rabbit monoclonal anti-γH2AX antibody (1:1000) for 2 h, followed with FITC-conjugated goat-anti-rabbit secondary antibody (1:500) for 1 h. Cells were counterstained with 4,6 diamidino-2-phenylindole (DAPI) for 15 min, washed with PBS and mounted. Fluorescence images were captured using an Olympus AX70 fluorescent microscope (Olympus, Tokyo, Japan). γH2AX foci in each cell (60–100 cells) was counted during the imaging process, and we used a setting as a standard for quantification in the selected cells to exclude relatively weak foci and background spots. In at least two independent experiments for each data point, samples were coded to prevent any bias.

### Statistical analysis

All values were expressed as mean ± standard deviation (SD) from at least three separate experiments. IC50 value was assessed by profit regression analysis. Student’s unpaired t-test, Mann–Whitney *U* test, chi-square test, log-rank statistic and Receiver Operating Characteristic (ROC) were performed using SPSS 21.0 statistical software (IBM). A two-tailed *P* value test was used in all analyses, and difference was considered as statistically significant if the *P* value was less than 0.05 (*P* < 0.05).

## Results

### Low expressions of miR-17 and miR-92 families are associated with cisplatin resistance

**Fig. 1:**
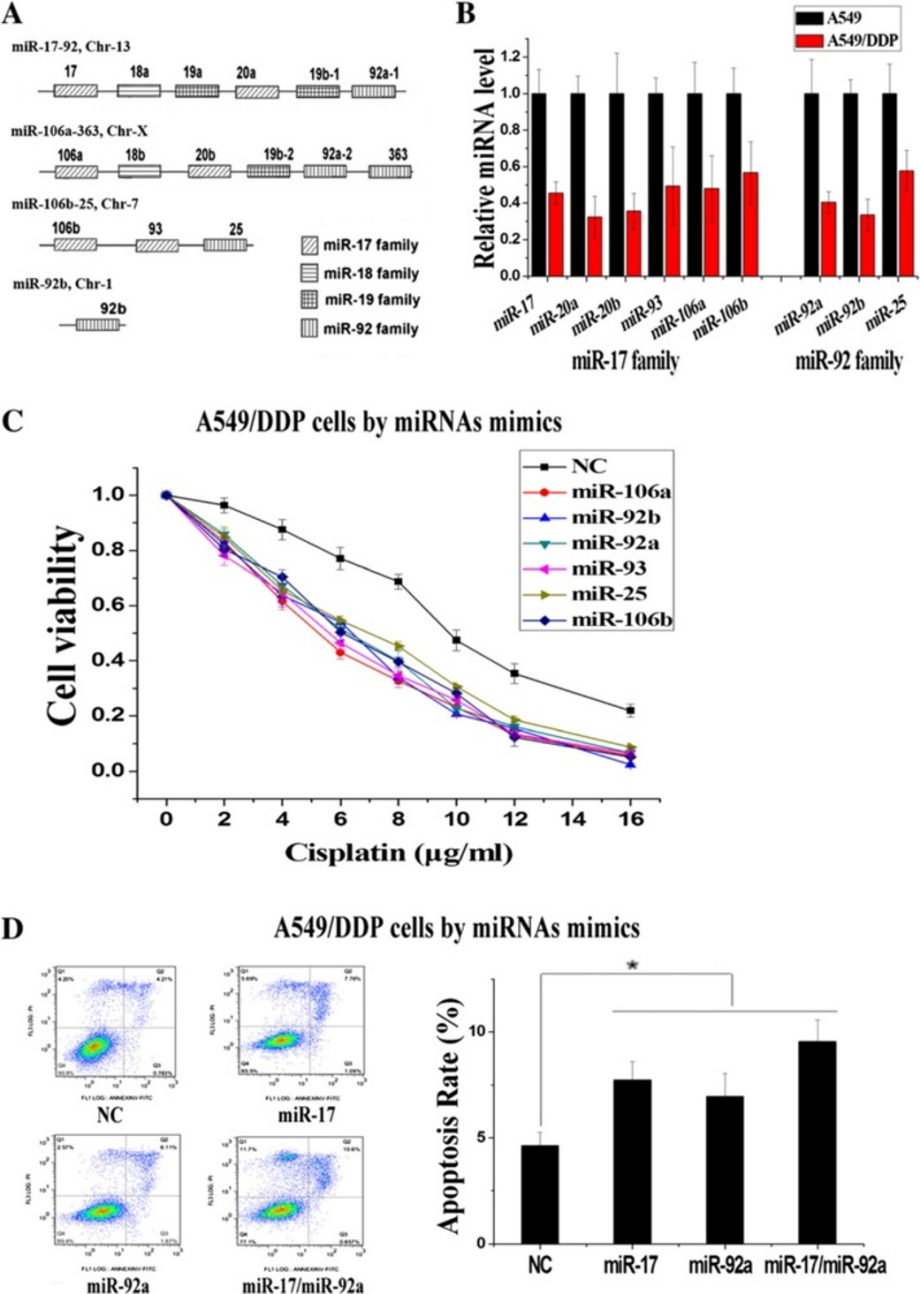
Both the miR-17 and miR-92 families were down-regulated in cisplatin resistance cells (**a**) Schematic representation of the four families of the miR-17 ~ 92 cluster and paralogous. miRNAs sharing the same seed sequence and involving in the same family were represented by boxes of the same pattern. **b** Down-regulation of miR-17 and miR-92 families in microarray experiments was validated by RT-PCR analysis. **c** Over-expression of different miRNA of miR-17 and miR-92 families decreased cisplatin resistance of A549/DDP cells significantly. **d** Apoptosis assays induced by cisplatin (2 μg/ml) were observed in different miRNA mimics treated A549/DDP cells. (*n* = 3, *P < 0.05)

Generally, miRNAs are produced from several genomic miRNA gene clusters and can be classified into different miRNA family based on the 6-nucleotide seed sequence similarities. For the miR-17 ~ 92 cluster and paralogous, there are 16 mature miRNAs which can be categorized into miR-17, miR-18, miR-19, and miR-92 families according to their conserved seed sequences (Fig. 1a). We have examined the expression levels of miRNAs in cisplatin-resistant cells (A549/DDP cells) and cisplatin-sensitive cells (A549 cells) using the microarray analysis assay in our previous study (20). It was found that 9 miRNAs of miR-17 ~ 92 cluster and paralogous (miR-20b, miR-20a, miR-17, miR-106a, miR-92b, miR-92a, miR-93, miR-25, miR-106b) were all down-regulated expressions in A549/DDP cells compared with A549 cells (Additional file 1: Table S1). Among these 9 miRNAs, 6 miRNAs belong to the miR-17 family and 3 miRNAs belong to the miR-92 family. Furthermore, the low expressions of miR-17 and miR-92 families in A549/DDP cells were further validated by using RT-PCR, and all of them were consistent with the microarray analysis results (Fig. 1b).

To confirm the regulatory functions of miR-17 and miR-92 families on cisplatin resistance, we altered the expressions of these miRNAs in A549/DDP cells by introducing miRNA mimics. As the regulatory functions of miR-17, miR-20a, miR-20b have been detected before, the remaining 6 miRNAs are detected in this study [20]. Over-expressions of miR-106a, miR-92b, miR-92a, miR-93, miR-25 and miR-106b restored sensitivity to cisplatin in A549/DDP cells, and the IC50 of cisplatin decreased from 9.71 ± 0.33 μg/ml to 5.17 ± 0.19 μg/ml (miR-106a), 6.08 ± 0.27 μg/ml (miR-92b), 6.25 ± 0.42 μg/ml (miR-92a), 5.53 ± 0.15 μg/ml (miR-93), 6.91 ± 0.36 μg/ml (miR-25) and 6.36 ± 0.45 μg/ml (miR-106b), as evidenced by the growth inhibition curve (Fig. 1c). Moreover, we examined the effects of miR-17 and miR-92 families on cisplatin-induced cellular apoptosis by using flow cytometric analysis. Based on the 6-nucleotide seed sequence similarities, we used miR-17 and miR-92a to represent these two families separately. Compared to the notarget control (NC), transfection of miR-17 or miR-92a mimics increased the cisplatin-induced cellular apoptosis in A549/DDP cells (Fig. 1d). These results suggested that low expressions of miR-17 and miR-92 families could maintain the cisplatin resistance in A549/DDP cells. Therefore, it is hypothesized that both miR-17 and miR-92 families play important roles in cisplatin resistance of NSCLC.

### Regulation of CDKN1A and RAD21 by miR-17 and miR-92 families

**Fig. 2:**
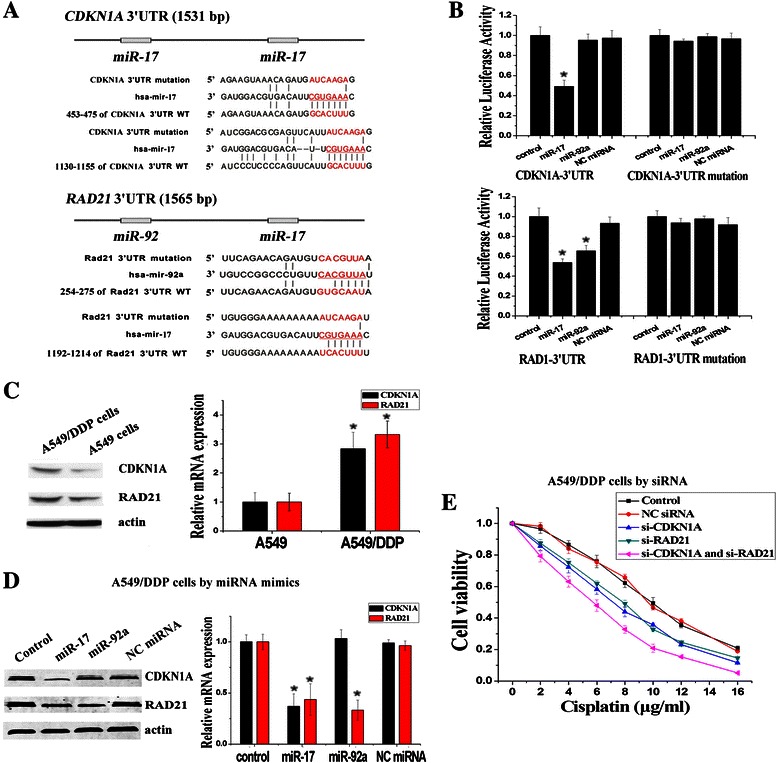
The miR-17 and miR-92 families regulated cisplatin resistance by targeting *CDKN1A* and *RAD21*. **a** Mutations were generated in the 3′-UTR sequence of *CDKN1A* and *RAD21* which located in the complementary site for the seed region of miR-17 and miR-92a as indicated. **b** Luciferase assays were used to observe the targeting effects of miR-17 and miR-92a on the 3′-UTR of *CDKN1A* and *RAD21*. **c** The protein and mRNA expression levels of CDKN1A and RAD21 in A549/DDP cells were significantly higher than those in A549 cells, detected by Western Blotting and RT-PCR assays. **d** The protein and mRNA expression levels of CDKN1A and RAD21 were decreased in A549/DDP cells after transfection of miR-17 and miR-92a mimics. **e** To varying degrees, individual or combined inhibition of *CDKN1A* and *RAD21* by siRNA decreased cisplatin resistance of A549/DDP cells significantly. (*n* = 3, *P < 0.05)

As miRNAs function by silencing target genes, we predict the targets of miR-17 and miR-92 families by using picTar and TargetScan prediction systems. In addition to *TGF*β*R2* (transforming growth factor beta receptor 2) which has been found in our previous study [20], we find that the 3′ untranslated region (3′-UTR) of *CDKN1A* contains two miR-17 family binding sites and 3′-UTR of *RAD21* contains one miR-17 family binding site and one miR-92 family binding site (Fig. 2a). In order to verify the putative target genes of miR-17 and miR-92 families by the dual-luciferase reporter assay, site-directed mutagenesis (MUT) were performed in the 3′-UTR of *CDKN1A* and *RAD21* (Fig. 2a). Compared with the MUT 3′-UTR control, miR-17 and miR-92a mimics could decrease the relative luciferase activity by binding to the target site in WT 3′-UTR of *CDKN1A* and *RAD21* (Fig. 2b). As shown in Fig. 2c and d, the expressions of CDKN1A and Rad21, which were all higher in A549/DDP cells compared with A549 cells, decreased significantly after the transfection of miR-17 mimics in A549/DDP cells. It was also observed that the expression of Rad21 decreased significantly after transfection of miR-92a mimics in A549/DDP cells (Fig. 2d). Furthermore, transfections of si-CDKN1A and si-RAD21 could reduce resistance to cisplatin in A549/DDP cells, and combined transfection of both si-CDKN1A and si-RAD21 could reduce resistance to cisplatin more significantly compared to transfection of single si-CDKN1A or si-RAD21 (Fig. 2e).

**Fig. 3:**
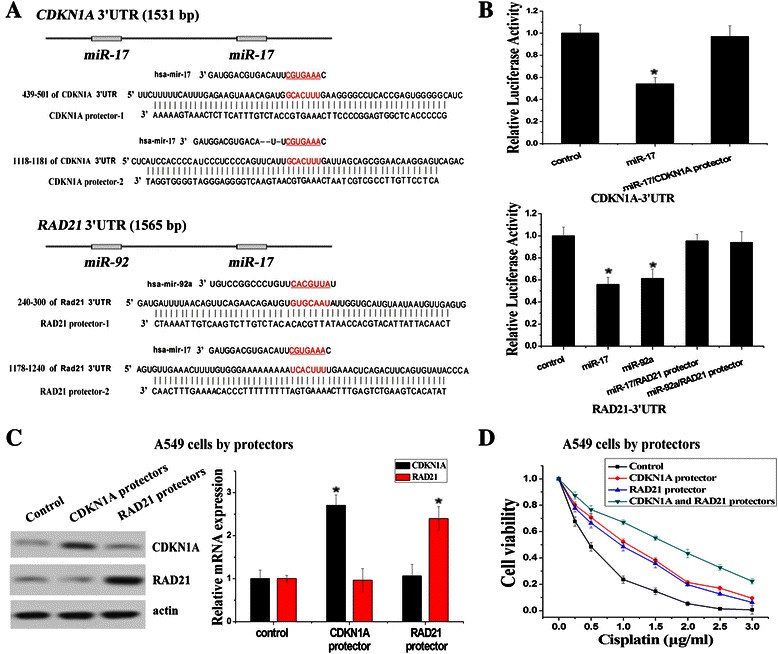
CDKN1A and RAD21 mRNA protectors specifically blocked the silencing effects of miR-17 and miR-92 families on the 3′-UTR of *CDKN1A* and *RAD21*. **a** CDKN1A and RAD21 mRNA protectors that could block the binding of miR-17 and miR-92a to their 3′-UTR were designed respectively. **b** CDKN1A and RAD21 mRNA protectors rescued the reduction of luciferase activities caused by the miR-17 and miR-92a targeting effect on their 3′-UTR respectively. **c** The protein and mRNA expression levels of CDKN1A and RAD21 were increased in A549 cells after transfection of CDKN1A and RAD21 mRNA protectors, detected by Western Blotting and RT-PCR assays. **d** To varying degrees, individual or combined transfection of CDKN1A and RAD21 mRNA protectors led to increased cisplatin resistance of A549 cells significantly. (*n* = 3, *P < 0.05)

To validate the targeting effect of miR-17 and miR-92 family on *CDKN1A* and *RAD21*, we use CDKN1A mRNA protectors and RAD21 mRNA protectors, which are complementary sequences that bind to the 3′-UTR of CDKN1A and RAD21 and block access to the binding sites of miR-17 and miR-92 family (Fig. 3a). The mRNA protector can compete with endogenous miRNAs for the binding site, with higher affinity to prevent miRNA/mRNA association [21]. As expected, CDKN1A and RAD21 protectors showed blocking effects on the silencing activities of miR-17 and miR-92a to *CDKN1A* and *RAD21* by luciferase assays (Fig. 3b). The transfection of CDKN1A and RAD21 protectors in A549 cells resulted in phenotypes similar to the up-regulation of CDKN1A and RAD21 and increased resistance to cisplatin (Fig. 3c and d). Same as the results in A549/DDP cells, combined transfection of both CDKN1A and RAD21 protectors also could increase resistance to cisplatin more significantly compared to transfection of single CDKN1A protectors or RAD21 protectors (Fig. 3d). These results suggested that *CDKN1A* and *RAD21* were two direct target genes of miR-17 and miR-92 families and the high expressions of CDKN1A and RAD21 could combinedly promote the cisplatin resistance in NSCLC.

### The inhibition of DNA synthesis as G1 phase arrest is regulated by targeting *CDKN1A*

**Fig. 4:**
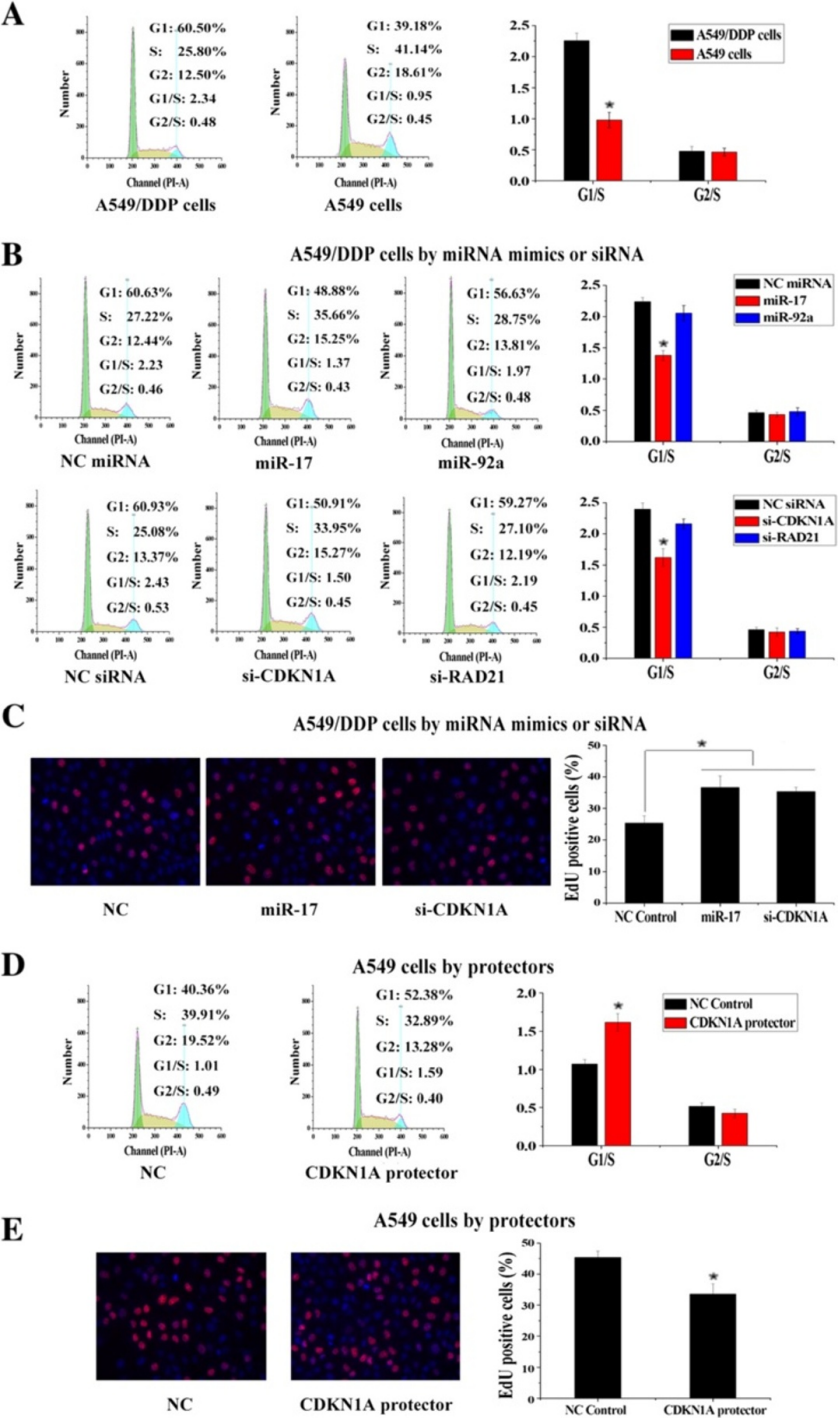
*CDKN1A* was a putative target gene in regulating G1 phase arrest and DNA synthesis. **a** Cell cycle of A549 cells and A549/DDP cells was analyzed with FACS. The bar graphs showed the relative quantity of G1/S and G2/S ratio. **b** Cell cycle analysis of A549/DDP cells transfected with miRNA mimics, siRNAs and NC controls. **c** EdU assay of A549/DDP cells transfected with miRNA mimics, siRNAs and NC controls. Hoechst stained cells were blue and EdU add-in cells were rad. The bar graphs showed the relative quantity of cells incorporating EdU. At least 300 cells were counted per well. **d** Cell cycle analysis of A549 cells transfected with mRNA protectors and NC controls. **e** EdU assay of A549 cells transfected with mRNA protectors and NC controls. (*n* = 3, *P < 0.05)

It has been showed that *CDKN1A* can act as a cell-cycle regulatory gene by inhibiting the activity of cyclin-CDK2 or cyclin-CDK4 complexes and blocking DNA synthesis [23]. Firstly, we compared the cell cycle status of A549 cells and A549/DDP cells by flow cytometry and found that A549/DDP cells were more arrest at G1 phase (>2-fold) (Fig. 4a). Then, in order to investigate whether the effect of miR-17 and miR-92 families on cisplatin resistance was related to cell cycle control, we analyzed the cell cycle of A549/DDP cells transfected with miR-17 mimics, miR-92a mimics, si-CDKN1A and si-RAD21. According to the results, both miR-17 mimics and si-CDKN1A could reduce G1 arrest (~0.6-fold) in A549/DDP cells (Fig. 4b). Our results indicated that low expression of miR-17 family in A549/DDP cells could trigger G1 phase arrest by targeting *CDKN1A*. We next conducted an EdU assay to detect the DNA synthesis of A549/DDP cells. EdU is a nucleoside analog of thymidine and is incorporated into DNA during active DNA synthesis, and the DNA synthesis (the proportion of cells incorporating EdU) can be directly measured by using EdU [22]. As shown in Fig. 4c, the transfection of miR-17 mimics or si-CDKN1A resulted in ~1.5-fold increase in the proportion of A549/DDP cells with DNA synthesis. We also transfected CDKN1A mRNA protectors in A549 cells, and as a result, more A549 cells were arrested at G1 phase (~1.5-fold) and the proportion of A549 cells with DNA synthesis also decreased significantly (~0.7-fold) (Fig. 4d and e). Our results supported the idea that low expression of miR-17 family could arrest cells at G1 phase and inhibit DNA synthesis by the specific regulation on *CDKN1A*.

### The repair of DNA damage is regulated by targeting *Rad21*

**Fig. 5:**
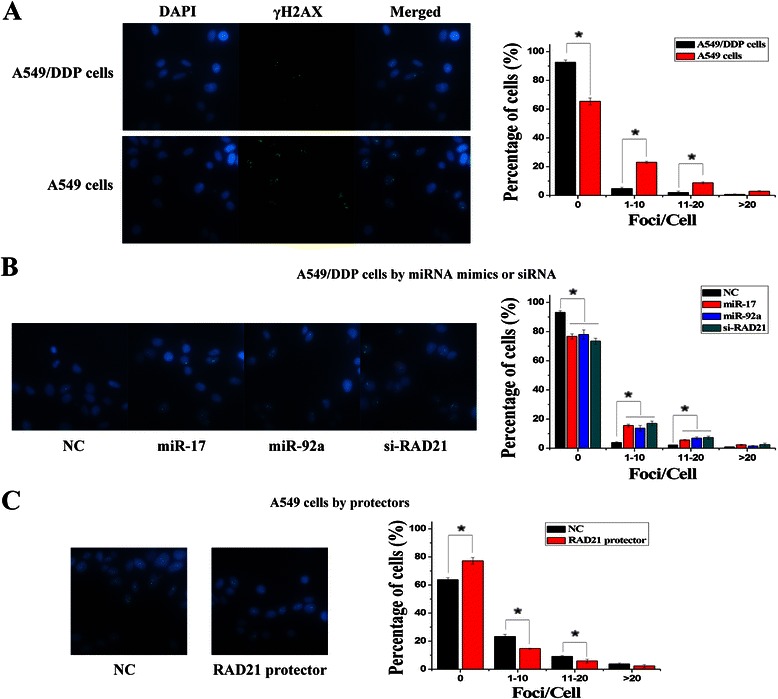
*RAD21* was a putative target gene in regulating DNA repair. **a** Representative images showed γH2AX foci induced by cisplatin treatment, and quantitative analyses showed more γH2AX foci in A549 cells than A549/DDP cells. **b** Quantitative analyses of γH2AX foci formation induced by cisplatin in A549/DDP cells transfected with miRNA mimics, siRNAs and NC controls. **c** Quantitative analyses of γH2AX foci formation induced by cisplatin in A549 cells transfected with mRNA protectors and NC controls. (*n* = 3, *P < 0.05)

*RAD21*, as a cohesin subunit, can regulate chromosome segregation in eukaryotes and is also involved in DNA repair through homologous recombination [24]. Therefore, we examined whether miR-17 and miR-92 families regulated DNA repair in A549/DDP cells by targeting *RAD21*. As phosphorylated histone H2AX (γH2AX) is an early participant in the cisplatin-induced DNA double-strand breaks (DSBs), we evaluated γH2AX foci formation assay to determine the degree of cisplatin-induced DNA damage. After cisplatin treatment, the cisplatin-induced γH2AX foci in A549/DDP cells were significantly lower than those in A549 cells (Fig. 5a). Moreover, transfections of miR-17 mimics, miR-92a mimics or si-RAD21 increased the cisplatin-induced γH2AX foci in A549/DDP cells, and transfection of RAD21 mRNA protectors reduced the cisplatin-induced γH2AX foci in A549 cells (Fig. 5b and c). It was indicated that low expressions of miR-17 and miR-92 families could promote the repair of cisplatin-induced DNA damage by increasing the expression of RAD21.

### The combined predictive function of miR-17 and miR-92 families on cisplatin resistance of NSCLC

**Fig. 6:**
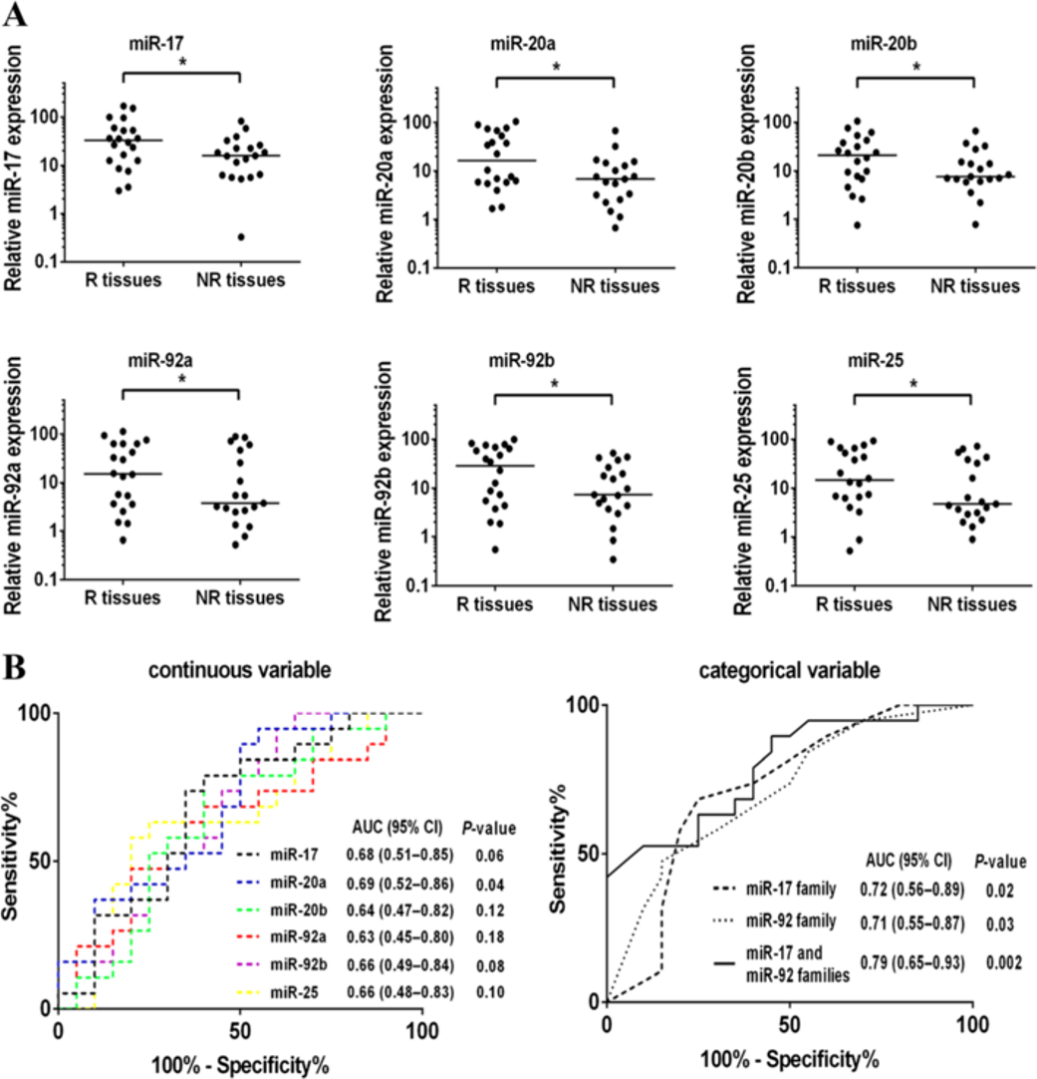
The expressions of miR-17 and miR-92 families were associated with the clinical outcome of platinum-based chemotherapy. **a** Scatter plots of expression levels of miR-17, miR-20a, miR-20b, miR-92a, miR-92b, miR-25 in responders and non-responders (NR, non-responder (stable or progressive disease); R, responder (response or partial response)). (*P < 0.05) **b** ROC analysis assessing the association the expressions of miR-17 and 92 families and the clinical outcome of platinum-based chemotherapy (NR or R) by miR-17, miR-20a, miR-20b, miR-92a, miR-92b, miR-25 levels as continuous variables, and combinations of miR-17, miR-20a, miR-20b, miR-92a, miR-92b, miR-25 levels using the sum of scores as categorical variables, where each miRNA was dichotomised and its categories represented by the score of 0 or 1 as follows: score 0 (low risk) = miR-17, miR-20a, miR-20b, miR-92a, miR-92b, miR-25 levels ≥ median; score 1(high risk) = converse of criteria for score 0

We compared the endogenous expressions of miR-17 and miR-92 families in platinum-based chemotherapy-resistant NSCLC tissues (NR tissues) and platinum-based chemotherapy-sensitive NSCLC tissues (R tissues) by RT–PCR. As shown in Fig. 6a, the analysis of multiple sets of comparable NR tissues and R tissues showed that three miRNAs of miR-17 family (miR-17, miR-20a and miR-20b) and three miRNAs of miR-92 family (miR-92a, miR-92b and miR-25) were less abundant in NR tissues than in R tissues significantly (*P* < 0.05). These results indicated that low expressions of miR-17 and miR-92 families were positively correlated with poorer therapeutic effect for NSCLC patients treated with platinum-based chemotherapy.

The potential of miR-17 and miR-92 families to predict the clinical outcome of platinum-based chemotherapy was assessed by ROC analysis. When analyzed as a continuous variable, the expression level of each miRNA of miR-17 and miR-92 families could predict the outcome of platinum-based chemotherapy (Fig. 6b). When analyzed as a categorical variable, the expression level of each miRNA was dichotomized and its categories represented by the score of 0 or 1, so that the combined predictive ability of each family (three miRNAs) or both two families (six miRNAs) could be assessed by using the sum of scores as variables. The combined score of both two families produced an AUC (Area Under the Curve) of 0.79 (95 % CI 0.65–0.93) which was higher than the score of individual family (Fig. 6b). Moreover, according to the correlation analysis between the expression levels of both two families (six miRNAs) and the clinicopathological factors of NSCLC patients, it was showed that the clinical outcome of cisplatin-based chemotherapy was significantly correlated with the expression levels of miR-17 and miR-92 families in NSCLC tissues (Table 1). These data indicated that a combined panel of miR-17 and miR-92 families could be used as potential biomarkers for predicting the clinical outcome of platinum-based chemotherapy in NSCLC.

**Table 1:** Correlation between the expressions of miR-17 and miR-92 families and the clinicopathological features of NSCLC patients

Clinicopathological factors		Expression level of 6 miRNAs^a^		χ^2^ value	*P* value
		High (*n* = 20)	Low (*n* = 19)		
Histological type	Adenocarcinoma	12	10	0.216	0.642
	Squamous carcinoma	8	9		
Gender	Male	14	15	0.407	0.523
	Female	6	4		
Age (years)	<60	9	7	0.265	0.607
	≥60	11	12		
Smoking	Nonsmoker	8	8	0.019	0.891
	Smoker	12	11		
TNM Clinical stage	IIIB	5	8	1.288	0.256
	IV	15	11		
Chemotherapy regimens^b^	DDP + TXT	9	6	0.745	0.689
	DDP + GEM	6	7		
	DDP + NVB	5	6		
Chemotherapy response^c^	CR + PR	7	13	4.366	0.036*
	SD + PD	13	6		

(* *P* < 0.05)

^a^Expression level of 6 miRNAs (miR-17, miR-20a, miR-20b, miR-92a, miR-92b, miR-25) was used the sum of scores as categorical variables, where each miRNA was dichotomised and its categories represented by the score of 0 or 1 as follows: score 0 (low risk) = miR-17, miR-20a, miR-20b, miR-92a, miR-92b, miR-25 levels ≥ median; score 1(high risk) = converse of criteria for score 0

^b^*DDP* cisplatin, *TXT* docetaxel, *GEM* gemcitabine, *NVB* vinorelbine

^c^*CR* complete response, *PR* partial response, *SD* stable disease, *PD* progressive disease

## Discussion

So far as we known, a lot of miRNAs have been implicated in a wide variety of malignancies, miR-17 ~ 92 cluster and paralogous are no exception [25–27]. Germline deletion of miR-17 ~ 92 cluster leads to perinatal death of mutant mice with defective development of the lung, heart and central nervous system, suggesting that miR-17 ~ 92 cluster and paralogous play important roles in many developmental processes [28–30]. It has been found that miR-17-92 cluster is continued overexpressed during cancer development and is recognized as a typical oncogene miRNA [31]. In lung cancer, high-expression of miR-17-92 cluster also has been found related to worse prognosis [32]. In this study, compared with cisplatin-sensitive A549 cells, the expressions of miR-17 and miR-92 families are inhibited in the cisplatin-resistant A549/DDP cells, suggesting that both miR-17 and miR-92 families play essential roles in cisplatin resistance. Normally, the functional studies of miRNAs need to focus on their target genes by which miRNAs regulate cell functions [33]. Each miRNA may potentially influence more than 100 target genes and regulate cancer network positively or negatively in a cellular context-dependent manner [34]. In addition to the identification of *TGF*β*R2* as a target gene for miR-17 family in the previous study [20], here, we also have validated *CDKN1A* and *RAD21* as two other target genes for miR-17 family and miR-92 family, revealing that miR-17 and miR-92 families form a complicated regulatory network together with their target genes in regulating cisplatin resistance.

In this study, we report that low expressions of miR-17 and miR-92 families can be contributing to cisplatin resistance by targeting *CDKN1A* and *RAD21*. The cyclin-dependent kinase inhibitor, CDKN1A, is a negative regulator of cell cycle progression in G1/S checkpoint and is sufficient to arrest the entry of G1 phase cells into S phase [35, 36]. CDKN1A protein can protect cancer cells from cisplatin-induced apoptosis as the DNA synthesis of S phase is reduced by G1 arrest, suggesting that one major reason of causing cisplatin resistance is that low expression of miR-17 family can block the DNA synthesis through CDKN1A-induced G1 arrest. Furthermore, RAD21 is an integral subunit of the cohesin complex involved in holding sister chromatids together after DNA synthesis until mitosis. Recent studies have suggested that the cohesin complex is also involved in the DNA repair by tethering sister chromatids and provided evidence that *RAD21* can promote the DNA repair of DSBs at G2 phase of cell cycle [37–39]. Therefore, low expressions of miR-17 and miR-92 families can enhance the RAD21-mediated DNA repair at G2 phase for reducing the cisplatin-induced DNA damage during S phase, resulting in causing cisplatin resistance. Our results indicate that miR-17 and miR-92 families may synergistically regulate cisplatin resistance in complex ways, as the regulatory function of their target genes can simultaneously inhibit DNA synthesis by G1 phase arrest and repair DNA damage at G2 phase.

**Fig. 7:**
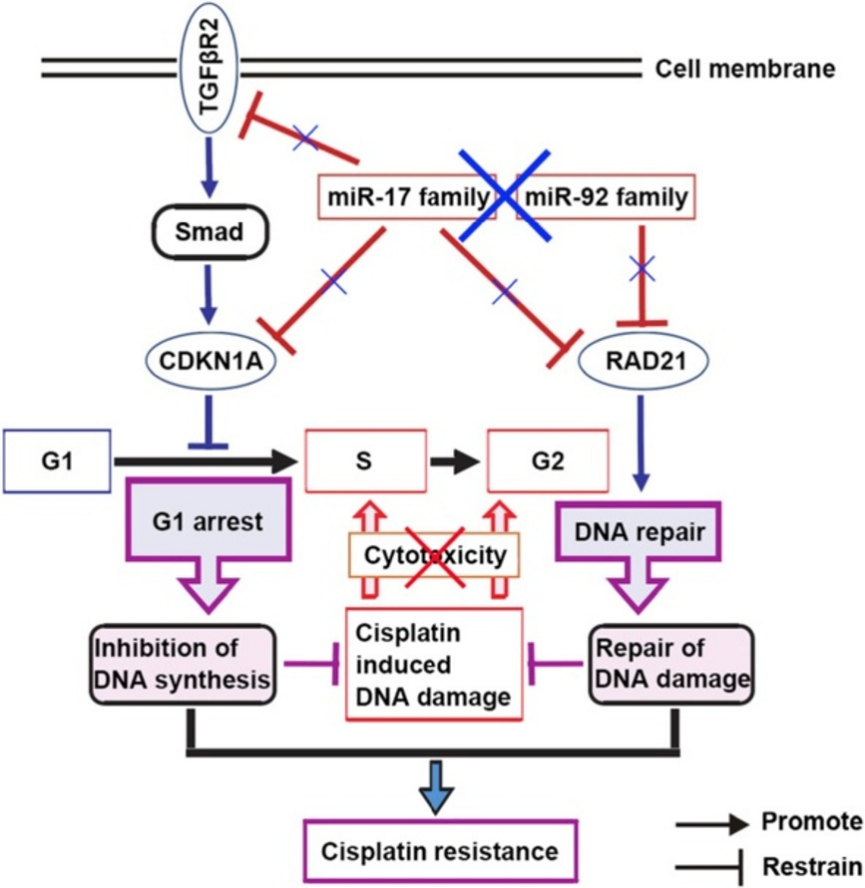
An illustrative summary of miR-17 and miR-92 families function on cisplatin resistance in NSCLC. miR-17 and miR-92 families function on the inhibition of DNA synthesis and the repair of DNA damage by repressing CDKN1A and RAD21

As a result of cisplatin-induced cytotoxicity in cancer cells, DNA damage are induced by the formation of DNA adducts during the process of DNA synthesis. Therefore, cisplatin-induced cytotoxicity can be decreased by the inhibition of DNA synthesis and the repair of DNA damage, and then cells stop cisplatin-induced programmed suicide and become resistant to cisplatin. Together with our previous results [20], it is demonstrated that low expressions of miR-17 and miR-92 families promote cisplatin resistance by acting on multiple key effectors along triggering the inhibition of DNA synthesis and the repair of DNA damage, and this is achieved in part by targeting *TGF*β*R2*, *CDKN1A* and *RAD21* (Fig. 7). On the one hand, high expressions of *TGF*β*R2* and *CDKN1A*, which exist in the same signaling pathway, can inhibit the cellular proliferation by arresting cells at G1 phase, resulting in the inhibition of active DNA synthesis at S phase and the reduction of cisplatin-induced DNA damage consequently. On the other hand, high expressions of *RAD21* can help cells to have more chances to repair cisplatin-induced DNA damage during S phase. Thus, under the cooperation of *TGF*β*R2*, *CDKN1A* and *RAD21*, the inhibition of DNA synthesis and the repair of DNA damage result in allowing cells to survival with the burden of potentially lethal cisplatin-induced cytotoxicity, and then cancer cells become resistant to cisplatin. Our results suggest that the low expressions of miR-17 and miR-92 families, which result in high expressions of TGFβR2, CDKN1A and RAD21, can synergistically inhibit DNA synthesis by induction of G1 phase arrest and reduce DNA damage by promotion of DNA repair, so that cancer cells maintain cisplatin resistance during chemotherapy.

We also identified that miR-17 and miR-92 families are significantly associated with the clinical outcome of platinum-based chemotherapy in NSCLC. Lower expression levels of miR-17 and miR-92 families in NSCLC tissues were observed in platinum-based chemotherapy-resistant patients compared with those in platinum-based chemotherapy-sensitive patients. Other study only assessed the higher levels of individual miR-92a-2* are associated with chemotherapy resistance and with decreased survival in patients with SCLC [40]. Here, compared with single miR-17 family or miR-92 family, the combined both of miR-17 and miR-92 families have more potential to be early therapeutic response biomarkers of platinum-based treatment in NSCLC. This discrepancy is likely due to the combined regulatory effects of miR-17 and miR-92 families in cisplatin resistance by simultaneously fine-tuning several genes.

## Conclusions

Overall, in the current scope of our work, it is suggested that miR-17 and miR-92 families can regulate cisplatin resistance through the synergistic and combined regulation of different targeted genes at the same time, such as *TGF*β*R2*, *CDKN1A* and *RAD21*. Although we just have studied two families of miR-17 ~ 92 cluster and paralogous here, it is expected that perhaps there are more targeted genes directly affected by other families, such as miR-18 family and miR-19 family. Therefore, all miR-17 ~ 92 families maybe participate in the control of their target genes and form complicated regulation networks together with their targeted genes to regulate chemotherapy resistance in NSCLC. Moreover, our study also reveals miR-17 and miR-92 families have the potential to predict the clinical outcome of platinum-based chemotherapy as molecular tumor biomarkers in NSCLC, and it is also suggested that miR-17 and miR-92 families can be used as potential therapeutic targets for reversing platinum-based chemotherapy resistance.

## Additional file

Additional file 1: Table S1. Differential miRNA expression in A549/DDP cells compared with A549 cells. (DOC 127 kb)

## Abbreviations

NSCLC: Non-small cell lung cancer
miR: microRNA
CDKN1A: Cyclin-dependent kinase inhibitor 1A
RAD21: Rad21 homolog (S. pombe)
DDP: Cisplatin
siRNA: Small interfering RNA
EdU: 5-ethynyl-2’-deoxyuridine
γH2AX: Phosphorylated histone H2AX
TGFβR2: Transforming growth factor beta receptor 2
DSBs: DNA double-strand breaks

## References

[1] Broxterman HJ, Gotink KJ, Verheul HM (2009). Understanding the causes of multidrug resistance in cancer: a comparison of doxorubicin and sunitinib. Drug Resist Updat.

[2] Stewart DJ (2010). Tumor and host factors that may limit efficacy of chemotherapy in non-small cell and small cell lung cancer. Crit Rev Oncol Hematol.

[3] Oguri T, Ozasa H, Uemura T, Bessho Y, Miyazaki M, Maeno K, Maeda H, Sato S, Ueda R (2008). MRP7/ABCC10 expression is a predictive biomarker for the resistance to paclitaxel in non-small cell lung cancer. Mol Cancer Ther.

[4] Rosell R, Skrzypski M, Jassem E, Taron M, Bartolucci R, Sanchez JJ, Mendez P, Chaib I, Perez-Roca L, Szymanowska A, Rzyman W, Puma F, Kobierska-Gulida G, Farabi R, Jassem J (2007). BRCA1: a novel prognostic factor in resected non-small-cell lung cancer. PLoS One.

[5] Saviozzi S, Ceppi P, Novello S, Ghio P, Lo Iacono M, Borasio P, Cambieri A, Volante M, Papotti M, Calogero RA, Scagliotti GV (2009). Non-small cell lung cancer exhibits transcript overexpression of genes associated with homologous recombination and DNA replication pathways. Cancer Res.

[6] Lee RC, Feinbaum RL, Ambros V (1993). The C. elegans heterochronic gene lin-4 encodes small RNAs with antisense complementarity to lin-14. Cell.

[7] Gregory RI, Shiekhattar R (2005). MicroRNA biogenesis and cancer. Cancer Res.

[8] Hatley ME, Patrick DM, Garcia MR, Richardson JA, Bassel-Duby R, van Rooij E, Olson EN (2010). Modulation of K-Ras-dependent lung tumorigenesis by MicroRNA-21. Cancer Cell.

[9] Calin GA, Croce CM (2006). MicroRNA signatures in human cancers. Nat Rev Cancer.

[10] Murphy BL, Obad S, Bihannic L, Ayrault O, Zindy F, Kauppinen S, Roussel MF (2013). Silencing of the miR-17 ~ 92 cluster family inhibits medulloblastoma progression. Cancer Res.

[11] Cardin R, Piciocchi M, Sinigaglia A, Lavezzo E, Bortolami M, Kotsafti A, Cillo U, Zanus G, Mescoli C, Rugge M, Farinati F (2012). Oxidative DNA damage correlates with cell immortalization and mir-92 expression in hepatocellular carcinoma. BMC Cancer.

[12] Valera VA, Walter BA, Linehan WM, Merino MJ (2011). Regulatory effects of microRNA-92 (miR-92) on VHL gene expression and the hypoxic activation of miR-210 in clear cell renal cell carcinoma. J Cancer.

[13] Rosell R, Lord RV, Taron M, Reguart N (2002). DNA repair and cisplatin resistance in non-small-cell lung cancer. Lung Cancer.

[14] He HT, Ni JD, Huang J (2014). Molecular mechanisms of chemoresistance in osteosarcoma. Oncology Letters.

[15] Sorrentino A, Liu CG, Addario A, Peschle C, Scambia G, Ferlini C (2008). Role of microRNAs in drug-resistant ovarian cancer cells. Gynecol Oncol.

[16] Donzelli S, Fontemaggi G, Fazi F, Di Agostino S, Padula F, Biagioni F, Muti P, Strano S, Blandino G (2012). MicroRNA-128-2 targets the transcriptional repressor E2F5 enhancing mutant p53 gain of function. Cell Death Differ.

[17] van Jaarsveld MT, Helleman J, Boersma AW, van Kuijk PF, van Ijcken WF, Despierre E, Vergote I, Mathijssen RH, Berns EM, Verweij J, Pothof J, Wiemer EA (2013). miR-141 regulates KEAP1 and modulates cisplatin sensitivity in ovarian cancer cells. Oncogene.

[18] Lee JH, Voortman J, Dingemans AM, Voeller DM, Pham T, Wang Y, Giaccone G (2011). MicroRNA expression and clinical outcome of small cell lung cancer. PLoS One.

[19] Lee YS, Dutta A (2009). MicroRNAs in cancer. Annu Rev Pathol.

[20] Jiang Z, Yin J, Fu W, Mo Y, Pan Y, Dai L, Huang H, Li S, Zhao J (2014). MiRNA 17 family regulates cisplatin-resistant and metastasis by targeting TGFbetaR2 in NSCLC. PLoS One.

[21] Zhang H, Shykind B, Sun T (2012). Approaches to manipulating microRNAs in neurogenesis. Front Neurosci.

[22] Salic A, Mitchison TJ (2008). A chemical method for fast and sensitive detection of DNA synthesis in vivo. Proc Natl Acad Sci U S A.

[23] Abbas T, Dutta A (2009). p21 in cancer: intricate networks and multiple activities. Nat Rev Cancer.

[24] Rainer PB, Suresh S (1992). Cloning and characterization of rad2l an essential gene of Schizosaccharomyces pombe involved in DNA double-strand-break repair. Nucleic Acids Res.

[25] Mi S, Li Z, Chen P, He C, Cao D, Elkahloun A, Lu J, Pelloso LA, Wunderlich M, Huang H, Luo RT, Sun M, He M, Neilly MB, Zeleznik-Le NJ, Thirman MJ, Mulloy JC, Liu PP, Rowley JD, Chen J (2010). Aberrant overexpression and function of the miR-17-92 cluster in MLL-rearranged acute leukemia. Proc Natl Acad Sci U S A.

[26] Chen ZL, Zhao XH, Wang JW, Li BZ, Wang Z, Sun J, Tan FW, Ding DP, Xu XH, Zhou F, Tan XG, Hang J, Shi SS, Feng XL, He J (2011). microRNA-92a promotes lymph node metastasis of human esophageal squamous cell carcinoma via E-cadherin. J Biol Chem.

[27] Kim K, Chadalapaka G, Lee SO, Yamada D, Sastre-Garau X, Defossez PA, Park YY, Lee JS, Safe S (2012). Identification of oncogenic microRNA-17-92/ZBTB4/specificity protein axis in breast cancer. Oncogene.

[28] Ventura A, Young AG, Winslow MM, Lintault L, Meissner A, Erkeland SJ, Newman J, Bronson RT, Crowley D, Stone JR, Jaenisch R, Sharp PA, Jacks T (2008). Targeted deletion reveals essential and overlapping functions of the miR-17 through 92 family of miRNA clusters. Cell.

[29] Shan SW, Lee DY, Deng Z, Shatseva T, Jeyapalan Z, Du WW, Zhang Y, Xuan JW, Yee SP, Siragam V, Yang BB (2009). MicroRNA MiR-17 retards tissue growth and represses fibronectin expression. Nat Cell Biol.

[30] Mendell JT (2008). miRiad roles for the miR-17–92 cluster in development and disease. Cell.

[31] Hayashita Y, Osada H, Tatematsu Y, Yamada H, Yanagisawa K, Tomida S, Yatabe Y, Kawahara K, Sekido Y, Takahashi T (2005). A polycistronic microRNA cluster, miR-17-92, is overexpressed in human lung cancers and enhances cell proliferation. Cancer Res.

[32] Ebi H, Sato T, Sugito N, Hosono Y, Yatabe Y, Matsuyama Y, Yamaguchi T, Osada H, Suzuki M, Takahashi T (2009). Counterbalance between RB inactivation and miR-17-92 overexpression in reactive oxygen species and DNA damage induction in lung cancers. Oncogene.

[33] Friedman RC, Farh KK, Burge CB, Bartel DP (2009). Most mammalian mRNAs are conserved targets of microRNAs. Genome Res.

[34] Krek A, Grün D, Poy MN, Wolf R, Rosenberg L, Epstein EJ, MacMenamin P, da Piedade I, Gunsalus KC, Stoffel M, Rajewsky N (2005). Combinatorial microRNA target predictions. Nat Genet.

[35] Ivanovska I, Ball AS, Diaz RL, Magnus JF, Kibukawa M, Schelter JM, Kobayashi SV, Lim L, Burchard J, Jackson AL, Linsley PS, Cleary MA (2008). MicroRNAs in the miR-106b family regulate p21/CDKN1A and promote cell cycle progression. Mol Cell Biol.

[36] Pickering MT, Stadler BM, Kowalik TF (2009). miR-17 and miR-20a temper an E2F1-induced G1 checkpoint to regulate cell cycle progression. Oncogene.

[37] Bauerschmidt C, Arrichiello C, Burdak-Rothkamm S, Woodcock M, Hill MA, Stevens DL, Rothkamm K (2010). Cohesin promotes the repair of ionizing radiation-induced DNA double-strand breaks in replicated chromatin. Nucleic Acids Res.

[38] Xu H, Balakrishnan K, Malaterre J, Beasley M, Yan Y, Essers J, Appeldoorn E, Tomaszewski JM, Vazquez M, Verschoor S, Lavin MF, Bertoncello I, Ramsay RG, McKay MJ (2010). *Rad21*-cohesin haploinsufficiency impedes DNA repair and enhances gastrointestinal radiosensitivity in mice. PLoS One.

[39] Yan M, Xu H, Waddell N, Shield-Artin K, Haviv I, McKay MJ, Fox SB, kConFab authors (2012). Enhanced *Rad21* cohesin expression confers poor prognosis in BRCA2 and BRCAX, but not BRCA1 familial breast cancers. Breast Cancer Res.

[40] Ranade AR, Cherba D, Sridhar S, Richardson P, Webb C, Paripati A, Bowles B, Weiss GJ (2010). MicroRNA 92a-2*: a biomarker predictive for chemoresistance and prognostic for survival in patients with small cell lung cancer. J Thorac Oncol.

