# Supplementary material for: The regulatory and predictive functions of miR-17 and miR-92 families on cisplatin resistance of non-small cell lung cancer
Source: BMC Cancer. 2015 Oct 19;15:731. doi: 10.1186/s12885-015-1713-z (PMC4617718; doi:10.1186/s12885-015-1713-z)
Supplement: Additional file 1: Table S1. — Differential miRNA expression in A549/DDP cells compared with A549 cells. (DOC 127 kb) [file 12885_2015_1713_MOESM1_ESM.doc]

**Additional file 1: Table S1.** Differential miRNA expression in A549/DDP cells compared with A549 cells.

| **miRNA ID** | **Fold Change (log2) (median)** | ***P* Value** |
| --- | --- | --- |
| hsa-miR-451 | -7.07 | <0.001 |
| hsa-miR-376c | 6.30 | <0.001 |
| hsa-miR-338-5p | -6.28 | <0.001 |
| hsa-miR-335 | -6.01 | <0.001 |
| hsa-miR-127-3p | 5.90 | <0.001 |
| hsa-miR-146a | 5.75 | <0.001 |
| hsa-miR-196a | -5.28 | <0.001 |
| hsa-miR-155 | -5.10 | <0.001 |
| hsa-miR-196b | -4.66 | <0.001 |
| hsa-miR-494 | 4.42 | <0.001 |
| hsa-miR-376a | 4.41 | <0.001 |
| hsa-miR-203 | -4.33 | <0.001 |
| hsa-miR-134 | 4.30 | <0.001 |
| hsa-miR-495 | 4.19 | <0.001 |
| hsa-miR-31 | 3.92 | <0.001 |
| hsa-miR-379 | 3.82 | <0.001 |
| hsa-miR-493* | 3.76 | <0.001 |
| hsa-miR-487b | 3.74 | <0.001 |
| hsa-miR-382 | 3.66 | <0.001 |
| **hsa-miR-20b** | **-3.65** | **<0.001** |
| hsa-miR-152 | -3.62 | <0.001 |
| hsa-miR-452 | -3.49 | <0.001 |
| hsa-miR-137 | 3.46 | <0.001 |
| hsa-miR-432 | 3.28 | <0.001 |
| hsa-miR-886-5p | -3.26 | <0.001 |
| hsa-miR-675 | -3.23 | <0.001 |
| hsa-miR-224 | -3.18 | <0.001 |
| hsa-miR-486-5p | -3.00 | <0.001 |
| hsa-miR-3178 | 2.94 | <0.001 |
| hsa-miR-1308 | -2.94 | <0.001 |
| hsa-miR-181d | -2.92 | <0.001 |
| hsa-miR-98 | -2.90 | <0.001 |
| hsa-miR-29a | 2.73 | 0.001 |
| hsa-miR-615-3p | -2.70 | <0.001 |
| hsa-miR-200b | 2.60 | <0.001 |
| hsa-miR-584 | -2.53 | <0.001 |
| hsa-miR-140-3p | -2.52 | <0.001 |
| hsa-miR-28-3p | 2.32 | <0.001 |
| hsa-miR-195 | 2.32 | <0.001 |
| hsa-miR-132 | -2.26 | <0.001 |
| **hsa-miR-20a** | **-2.22** | **<0.001** |
| hsa-miR-431 | 2.22 | 0.001 |
| **hsa-miR-17** | **-2.22** | **<0.001** |
| **hsa-miR-106a** | **-2.22** | **<0.001** |
| hsa-miR-34a | 2.15 | 0.001 |
| hsa-miR-574-3p | -2.11 | 0.001 |
| hsa-miR-10a | -2.06 | 0.001 |
| hsa-miR-221 | 2.04 | 0.001 |
| hsa-miR-193b* | -2.04 | 0.001 |
| hsa-miR-28-5p | 2.02 | 0.001 |
| hsa-miR-744 | 2.01 | 0.001 |
| hsa-miR-151-3p | 2.00 | 0.001 |
| hsa-miR-424 | -1.97 | 0.001 |
| hsa-miR-27b | 1.94 | 0.002 |
| hsa-miR-625 | 1.90 | 0.002 |
| hsa-miR-4298 | -1.89 | 0.002 |
| hsa-miR-1469 | 1.87 | 0.002 |
| hsa-miR-183 | 1.86 | 0.002 |
| hsa-miR-320e | -1.82 | 0.003 |
| hsa-miR-574-5p | -1.77 | 0.003 |
| hsa-miR-483-5p | -1.74 | 0.003 |
| hsa-miR-424* | -1.63 | 0.003 |
| hsa-miR-200c | -1.62 | 0.003 |
| **hsa-miR-92b** | **-1.60** | **0.004** |
| hsa-miR-320d | -1.58 | 0.006 |
| hsa-miR-3141 | -1.57 | 0.005 |
| **hsa-miR-92a** | **-1.56** | **0.005** |
| hsa-miR-3195 | 1.55 | 0.005 |
| hsa-miR-324-5p | 1.53 | 0.005 |
| hsa-miR-107 | 1.52 | 0.006 |
| hsa-miR-27a | 1.51 | 0.006 |
| hsa-miR-374b | -1.44 | 0.007 |
| hsa-miR-3172 | 1.42 | 0.008 |
| hsa-miR-103 | 1.41 | 0.007 |
| hsa-miR-130a | 1.38 | 0.008 |
| hsa-miR-7 | -1.38 | 0.007 |
| hsa-miR-2861 | -1.35 | 0.007 |
| hsa-miR-151-5p | 1.34 | 0.008 |
| hsa-miR-320b | -1.32 | 0.008 |
| hsa-miR-1975 | -1.30 | 0.007 |
| hsa-miR-455-3p | 1.25 | 0.007 |
| hsa-miR-320a | -1.18 | 0.007 |
| hsa-let-7b | -1.17 | 0.008 |
| hsa-miR-222 | 1.17 | 0.007 |
| hsa-miR-629 | -1.16 | 0.007 |
| hsa-miR-877 | -1.16 | 0.007 |
| hsa-let-7e | -1.15 | 0.008 |
| hsa-miR-320c | -1.15 | 0.007 |
| hsa-miR-762 | 1.15 | 0.007 |
| hsa-miR-100 | 1.14 | 0.007 |
| hsa-miR-192 | 1.13 | 0.008 |
| hsa-miR-21 | 1.12 | 0.008 |
| hsa-miR-4281 | 1.10 | 0.008 |
| **hsa-miR-93** | **-1.06** | **0.007** |
| hsa-miR-30b | -1.00 | 0.008 |
| hsa-miR-182 | 1.00 | 0.007 |
| hsa-miR-181a | 0.96 | 0.008 |
| hsa-miR-1979 | -0.94 | 0.008 |
| **hsa-miR-25** | **-0.93** | **0.008** |
| hsa-miR-24 | 0.88 | 0.009 |
| hsa-miR-125b | 0.88 | 0.009 |
| hsa-miR-30a* | -0.88 | 0.009 |
| hsa-miR-454 | 0.87 | 0.01 |
| hsa-miR-128 | 0.83 | 0.01 |
| hsa-let-7i | 0.80 | 0.011 |
| hsa-miR-23b | 0.68 | 0.015 |
| hsa-miR-361-5p | -0.66 | 0.016 |
| hsa-miR-15b | 0.64 | 0.017 |
| hsa-let-7c | -0.63 | 0.025 |
| hsa-miR-423-5p | -0.63 | 0.023 |
| hsa-miR-1246 | 0.63 | 0.027 |
| hsa-miR-23a | 0.61 | 0.026 |
| hsa-miR-16 | 0.60 | 0.031 |
| hsa-miR-193a-5p | -0.59 | 0.033 |
| hsa-miR-1268 | -0.55 | 0.037 |
| hsa-miR-1915 | 0.53 | 0.038 |
| **has-miR-106b** | **-0.49** | **0.032** |
| hsa-miR-638 | 0.37 | 0.041 |
| hsa-miR-720 | 0.25 | 0.043 |
| hsa-miR-26b | -0.23 | 0.041 |
| hsa-miR-26a | 0.19 | 0.045 |
| hsa-let-7d | -0.17 | 0.049 |

Note: Negative values represent down-regulation; positive values represent up-regulation.
